# Letters of condolence: assessing attitudes and variability in practice amongst oncologists and palliative care doctors in Yorkshire

**DOI:** 10.3332/ecancer.2016.642

**Published:** 2016-05-18

**Authors:** Jessica S Hayward, Oluwatobi Makinde, Naveen S Vasudev

**Affiliations:** 1 School of Medicine, University of Leeds, Leeds, LS2 9JT, UK; 2 Leeds Institute of Cancer and Pathology, University of Leeds, LS9 7TF, UK; 3 St James’s Institute of Oncology, Leeds, LS9 7TF, UK

**Keywords:** letters, condolence, oncologists, palliative care, bereavement

## Abstract

**Background:**

Following a patient’s death, some doctors routinely write a letter of condolence to the bereaved family. Practice appears to vary widely but this is poorly documented, particularly in the UK setting. We wished to explore the attitudes of oncologists and palliative care consultants towards writing letters of condolence to patient’s families.

**Methods:**

A sample of oncology and palliative care consultants from across Yorkshire were invited via email to complete an anonymous online survey. The survey aimed to identify current practice regarding condolence letter writing and respondents attitudes towards this.

**Results:**

A total of 47 (72%) recipients completed the survey, comprised of clinical oncologists (45%), medical oncologists (42%), and palliative care consultants (13%). The majority (87%) reported sending condolence letters, but amongst this group, only 49% indicated they do this ‘often’ or ‘always’. When asked whether they would use a standard template letter, should it be made available, 77% of participants responded negatively. Many later commented that a template with room for flexibility would be better received. The majority (72%) were also not in favour of the introduction of policies to try to unify practices.

**Conclusions:**

Practices and attitudes towards condolence letter writing are variable. The participants in this study felt strongly about when and how they wished to express condolences. A single unifying policy seems unlikely to be appropriate or feasible.

## Background

Cancer causes more than a quarter of all deaths in the UK [1]. The national cancer agenda is focused on improving provision of good end-of-life care, as evidenced by the *Achieving world-class cancer outcomes: a strategy for England 2015–2020* Cancer Taskforce report and the recently issued National Institute for Health and Care Excellence *Care of dying adults in the last days of life* guidance.

Less attention has been paid to the care of the bereaved family, following a patient’s death. The loss of a loved one is often a very difficult and sensitive time, yet support during this period of grief is not always considered. It has been suggested that the responsibility of healthcare professionals does not end with the death of a patient, but rather extends to also helping the bereaved family [2–4]. Palliative care guidelines from the US National Comprehensive Cancer Network [5] and the American College of Chest Physicians [6] both recommend communicating with family and friends after death by way of a card, letter, or call. Expressions of condolence may provide comfort to bereaved relatives [7–9], whereas it can lead to disappointment and feelings of abandonment in the absence of any form of contact [2, 10, 11]. Furthermore, doctors themselves may experience grief over the deaths of patients and engaging in bereavement practices may have a positive impact [12].

Very few studies have examined current bereavement practices of healthcare professionals, particularly within a UK setting. In the current study, we examined the bereavement practices of oncologists and palliative care consultants in Yorkshire and their attitudes towards this, with a particular focus on the use of condolence letters.

## Methods

### Study design and participants

The study took the form of an anonymous online survey sent to palliative care and oncology consultants based at one cancer centre and four cancer units across Yorkshire. We approached clinical and medical oncologists, who are both trained in the care of the full range of malignant diseases. Clinical oncologists, however, are the only specialist group trained in the treatment of patients with radiotherapy, whilst medical oncologists in particular specialise in the administration of systemic therapies.

The use of an online survey was favoured over other data collection techniques based on a number of practical reasons. Yorkshire is a large area, such that travelling to interview doctors or hold focus groups would not have been feasible. Asking doctors to complete a short online survey in their own time, in a location of their choice, at no expense such as travel or postage, seemed the most appropriate option and offered the best way to maximise response rate.

**Figure 1.**
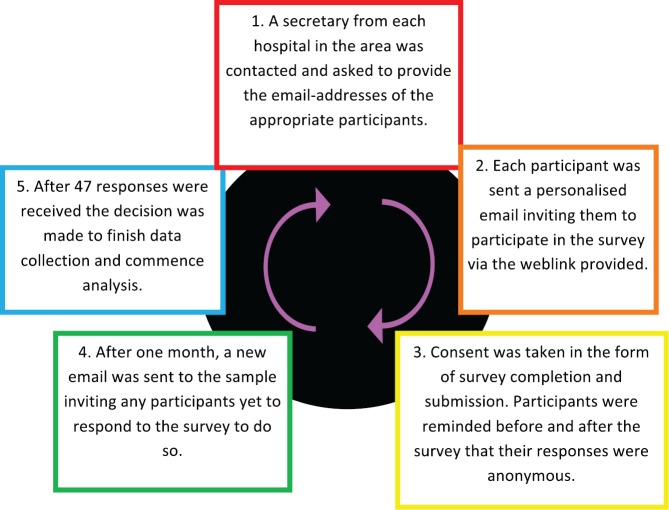
Study outline.

The study outline is shown in Figure 1. Participants were initially approached via email in January 2015, which included a brief description of the study and included a link to complete an anonymous online survey. A second reminder email was issued one month later. Participants provided consent electronically by agreeing to take part in the study and completing the survey. No incentive was offered for participation.

### Questionnaire

A questionnaire was constructed and administered using an online tool (Bristol Online Surveys (www.onlinesurveys.ac.uk)). All participants were asked for basic demographics (specialty, gender, years in practice) before being asked whether they send letters of condolence. Participants who answered ‘yes’ were then asked a series of follow-up questions to understand more about their practice, including frequency, timing, and style of letter writing. Those participants who said they did not send condolence letters were directed to a separate set of questions, exploring their reasons for this, and whether they employ any alternative measures. Finally, all participants were asked whether they would support a policy to unify practices in terms of condolence letter writing.

### Data analysis

The survey collected both categorical and free-text response data. Descriptive statistics were used to compare the frequency of responses. Free-text comments were reviewed together with the categorical responses to help explain the reasoning behind some of the behaviours.

## Results

### Participant demographics

In total, 47 (72%) of the 65 consultants who were approached responded to the survey. The sample, 55% of whom were male, consisted of 21 (45%) clinical oncologists, 20 (42%) medical oncologists, and 6 (13%) palliative care consultants. The majority of participants (26; 55%) had been in a consultant position for over ten years, 11 (23%) had been a consultant for 6–10 years, and 10 (21%) had been a consultant for 1–5 years.

### Current practices

Amongst all participants, 35/47 (75%) indicated that they sent letters of condolence to families following the death of a patient. We then asked these participants to answer a series of follow-up questions to explore their practice further.

### Participants who wrote condolence letters

We first asked about the frequency with which participants wrote condolence letters. Here, 41/47 (87%) participants responded that they occasionally (21/41; 51%), often (13/41; 32%) or always (7/41; 17%) send condolence letters. This is higher than the 35/47 participants who initially answered ‘yes’, suggesting that participants may have interpreted the initial question differently.

**Figure 2.**
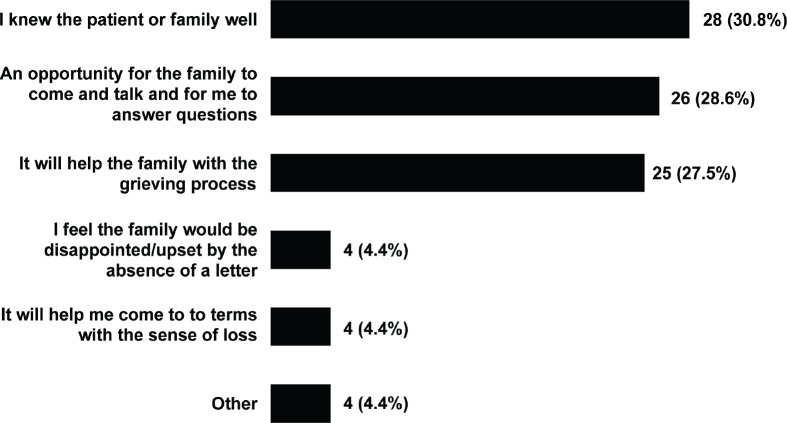
Reasons for writing condolence letters. Participants could select more than one option.

We next asked for the reasons that participants write condolence letters (Figure 2). Participants were asked to choose as many reasons as they felt applied, as well given the opportunity for free-text responses. The most common reasons selected were ‘knowing the patient or family well’ (31%), ‘helping the family to grieve’ (28%), and ‘giving the family the opportunity to talk and ask questions about their relative’ (29%). Other reasons that were expressed included:

*‘An opportunity to provide missing information to family members, to explain what happened and why. Possibly to avoid complaints by correcting misunderstandings’*.

*‘I think it is polite and professional to acknowledge their loss’*.

*‘It is a simple mark of respect to the family and the deceased. It allows the family to return, if wanted, to discuss any issues around the death’*.

Participants were then asked about the style and content of their letters. When asked whether they used a standard template, only 12/40 (30%) answered ‘yes’, suggesting that the majority routinely individualise their content. Amongst those that used a standard template, we asked from them examples of the sort of wording they used. In general, participants expressed their sympathy to the family regarding the loss of their loved one and finished by offering the opportunity to arrange a meeting to answer any questions in the future. For example:

Dear…, I was sorry to hear the sad news of the recent death of … Please accept my sincere condolences, and those of the team here at… You may find, as time goes by, that you have questions or issues that you would like to discuss with me. If you would like to do this, please contact my secretary….

Others indicated that they start with a template, but may then change it.

When producing letters, 31/39 (79.5%) participants indicated that they type rather than handwrite their letters, and 32/40 (80.0%) used trust headed paper, with few using alternatives such as condolence cards (17.5%) or non-headed paper (2.5%).

We next asked about the timing of when letters were sent. The majority (24/29; 61.5%) indicated that they do this 3–6 weeks after the patient’s death, with the remainder sending them within two weeks. No participants waited beyond six weeks. Participants were then asked why they sent letters at this time. Many views were expressed. Some indicated that they do this as soon as possible, for example:

‘Any later than 2 weeks may stir things up/aggravate grief. The letter is supposed to help’,

‘I send them as soon as I hear that a patient has died’.

Others, however, felt that waiting a short time was preferential:

‘So it’s not too upsetting but still early enough to give them a chance to talk’.

‘Allow some time for the family’.

‘The family need to go through the funeral and immediate shock period, come to terms and then be thinking more clearly about whether they want to come and chat again to the team rather than too soon after bereavement..’

A number of participants described variability in the timing, dependent on finding time, and also when they are informed of the patient’s death, which in itself may vary dependent on whether the death occurred in or out of hospital, for example.

Finally, we asked if participants used any other types of bereavement practice, besides condolence letters. A telephone call was used by 4/42 (9.5%) and 1/42 (2.4%) would visit the family at home. None of the participant said that they attend patient’s funeral. Those who used other practices spoke of another member of the healthcare team contacting the family and providing support, such as the team’s cancer nurse specialist.

### Participants who do not send letters of condolence

Participants who indicated that they do not send condolence letters were directed to a separate section of the questionnaire. When asked whether condolence letter writing was something they had never done, 8/11 (72.7%) said this was the case. The remainder indicated that they used to, but now have ceased doing so. The reasons for this were, in one case the participant was now too busy. In the other two cases the reasons were as follows, and suggest that working within a team may mean others carry out this role:

*‘When I was based on the inpatient unit I would send a standard letter. I am now community based so families receive follow up from the nurse specialist, either by phone, card, or in person. I therefore rarely duplicate this’*.

*‘Previously I basically had a single handed practice and saw all my patients myself. Now I work as part of a team and may have not seen patients for a while before they pass away’*.

**Figure 3.**
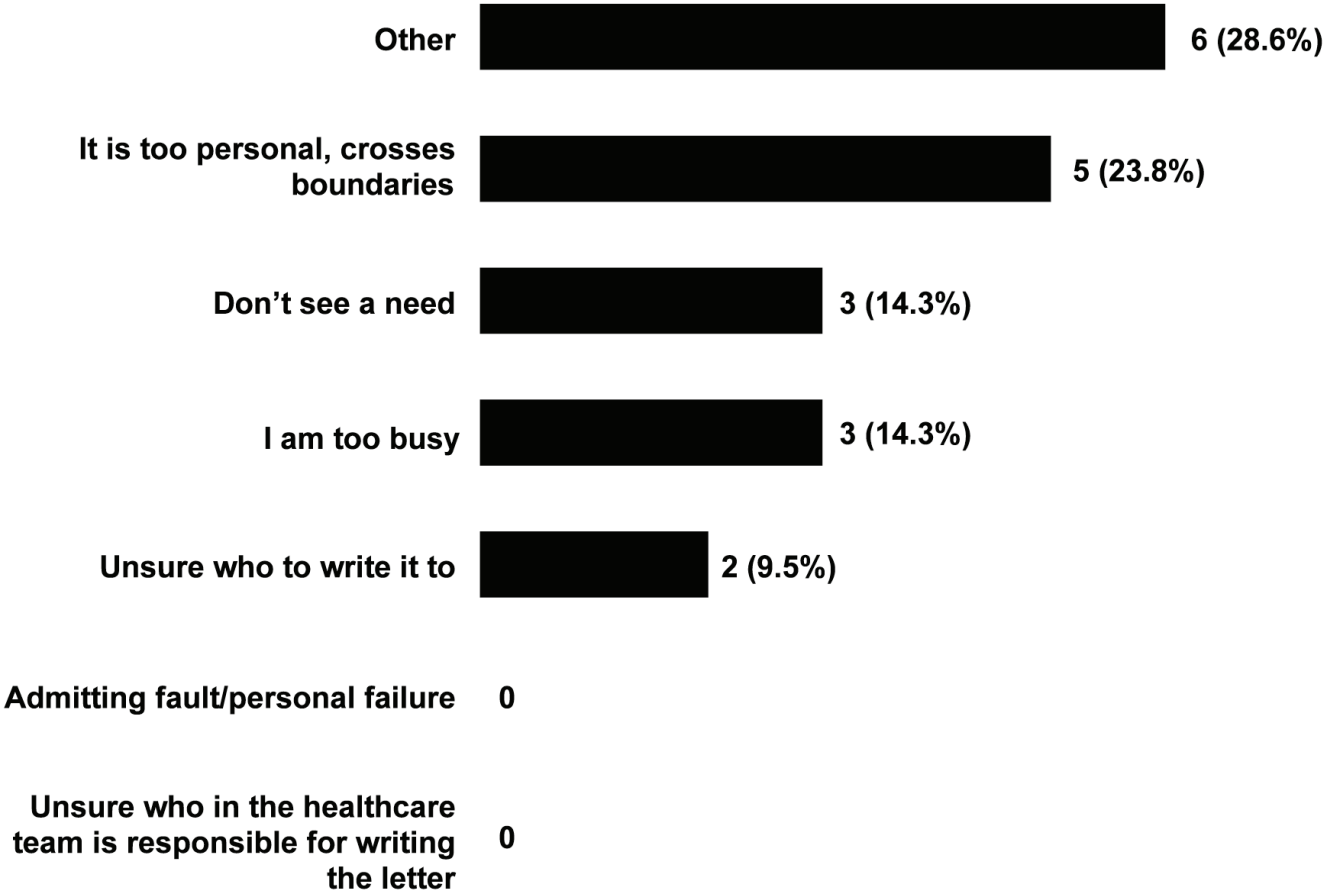
Reasons for not sending letters of condolence. Participants could select more than one option.

Beyond this, other reasons for not writing condolence letters are shown in Figure 3. Again, participants could select more than one answer, as well as being able to provide free-text responses.

The most common reasons were that it felt too personal and crossed boundaries, being too busy, or that there was no need. For example, one participant explained:

‘Death is a predefined outcome in our palliative patients and global use of a condolence letter seems unjustified’.

Others expressed that they were concerned a letter may not be helpful and could in fact cause upsets:

‘*Many patients die at home and we do not know the exact circumstances. It is possible that if it was not a ‘peaceful’ death, that the condolence letter could seem insensitive’.*

‘Potential to upset relatives who might have a wide range of opinions of the service provided by the healthcare team’.

Alternatives to letters of condolence were again not widely employed amongst participants, with 3/14 (21.4%) making a telephone call, and 1/14 (7.1%) either visiting the family or attending the funeral.

## Future practice

Lastly, we asked all participants about whether they would support a policy that sought to unify practice in terms of condolence letter writing. The majority of respondents (34/47; 72.3%) indicated they would not support such a policy. In addition, we asked if participants would be prepared to use a set template, which results in an identical letter, besides the name of the deceased, being distributed to families. Again, the majority (36/47; 76.6%) indicated they would not be prepared to do so.

These questions were intended to be provocative and indeed proved to be highly contentious. All respondents (47/47) chose to expand on the reasons behind their choice of answers using the free-text option. Several key themes emerged. Almost one half of the respondents (22/47) used words such as (im)personal or individual in reference to a standard template and many felt very strongly on this issue. Typical responses included:

‘*This should be a personal letter in the same way every clinic letter is personal. An automated letter for me would have no value and if I received one I might feel insulted’.*

‘*I think this is an awful idea; the whole point of a condolence note is that it be personal and relevant. A standard, typed pro-forma would be worse than nothing. It smacks of mass production’.*

‘It has to be personal!! I feel very strongly about this as I believe this should not be like any other letter from hospital. Misses the point altogether’.

For some participants, however, a template seemed to provide convenience and they supported the idea, particularly if it allowed the opportunity for it to be used as a starting point that could be modified.

‘*We use a more or less standard letter in our team but if there was a departmental standard I would consider using it’.*

‘It would depend on the details. I do modify my letters on occasions but I find that I am trying to say the same thing to most people and so even if we did not use a template I would expect to end up using similar phrases’.

‘Efficient – I would want the ability to adapt the standard template’.

Few participants commented further on the idea of unifying practice. The majority had indicated they would not support such a policy.

‘*I think it is impossible to enact a unifying practice. If I was in paediatric oncology, which is pretty intense, I would probably write bereavement letters and similarly with young adults. Lung cancer is different, a letter (particularly a standard one) would seem crass and inappropriate after one has attended a patient through the final days of their life’.*

‘This is a matter of professional discretion and judgement and should not become a ‘policy’.

One participant indicated their support but still qualified this.

‘*I think a unified practice–i.e. an agreement that we all send letters—would be a good thing. However, I think it would have a negative impact if one family, losing two members, received an identical letter from a different consultant after the second death. So I would encourage and help consultants to compose their own “standard” letter and also to personalise letters whenever possible’,*

## Discussion

Little is currently known regarding contemporary bereavement practices amongst cancer physicians in the UK. Here, we show that amongst the participants in our sample, the majority engage in writing letters of condolence at least occasionally (41/47 (87%)). This was by far the commonest method of contact. The proportion of participants making telephone calls was generally low, but was higher amongst those who reported not sending condolence letters (9.5% versus 21.4%). Only one participant indicated they attended patient’s funeral.

Previous studies report the proportion of participants who always or usually express some form of condolence to the family of a deceased patient between 55–74% [13–16]. In comparison, overall 42.5% of participants in the current study reported always or often writing letters of condolence. This is a lower figure than in previous studies although there are numerous possible explanations for this. Firstly, our study focused on the use of condolence letters. We did not ask participants who made telephone calls whether this is something they usually or always did, but it is possible this may account for some of the difference. Secondly, the reported studies have varying representation of medical and clinical oncologists and palliative care physicians, which may influence the results [13, 15]. Thirdly, these studies were conducted in the US, Canada, and Israel and may therefore reflect differences in service structure as well as cultural and religious differences. Finally, some studies were conducted almost a decade ago [15]. Working patterns and caseload are likely to have changed over this time and the establishment of multidisciplinary teams with delegation of tasks as well as changes in attitudes towards bereavement practices over time may all influence the data.

Over half of participants in our study who said they wrote letters of condolence indicated they only do this occasionally. What determines when doctors feel they should write? The most likely explanation is how well the doctor knew the patient and their family. Knowing the patient well was the commonest reason (30.8%) given for why a letter was written in this study. Amongst 126 Israeli cancer specialists, respondents were much more likely to visit the family or attend memorial services/funerals if they had a special bond with the patient and their family (29% versus 45%), and less likely to simply write a letter or make a telephone call (45% versus 33%) [14]. Thus, doctors may only contact bereaved relatives if there was strong relationship between themselves and the patient [17, 18].

The ideal content of condolence letters has been described by others and should typically acknowledge the loss, express sympathy, note special qualities, or a personal memory of the deceased, offer to remain available for support or to review the management of the disease, and end with an expression of sympathy [2, 4, 19]. A third of participants in this study used a standard template, which appeared to contain many of these elements, but that may not necessarily be personalised in the manner suggested. Others indicated that they may start with a template but then alter it appropriately, a practice commonly reported elsewhere [17]. It is unclear if participants who choose to use a template were also more likely to be those who always send condolence letters. It is likely that a balance must be found between the desire to reach out to all bereaved relatives and finding the time and knowing the patient well enough to write a truly personal note.

The optimal time to send a condolence letter is not known. Little agreement was found in the current study, with some suggesting this should be done as soon as possible and others preferring to wait 3–6 weeks. Most participants typed their letters (79.5%) and used hospital trust headed paper (80%). We did not explore the reasons for this preference but potentially this may simply be more efficient or may be felt to be too personal/crosses boundaries to do otherwise. In a survey of 432 US physicians, nurse practitioners, and physician assistants, including all specialities (i.e. not limited to oncologists), approximately 40% of participants sent a handwritten condolence note or commercial card, with only 5% using a typed note on letterhead [16]. The reason for this large discrepancy is uncertain but may lie in the wide distribution of medical specialities included, with physicians presumably experiencing vastly different rates of patient’s death. In either case, the content is arguably more important than the manner in which it is presented [4].

The strongest views expressed in this study related to the question of introducing a standardised template for all condolence letters. Little support was provided for this and, at best participants felt this was only acceptable if it could then be individually altered. The majority (72.3%) of participants were also against the introduction of a policy that sought to unify practices. Amongst the views expressed it was felt that this was a matter of professional discretion and judgement and should not become a policy*.* Others have reported similar findings with participants suggesting this is a personal behaviour related to the connection with patients and that institutional policies could lessen their impact [16]. Granek and colleagues suggest that a standardised, non-mandatory protocol may be helpful, with condolence cards made available in the department and time set aside each week for making calls or writing letters [12].

This study has several limitations. The sample size was relatively small and the study provides the views of oncologists and palliative care doctors from just one region in the UK. Furthermore, the study was limited to oncologists and palliative care physicians. We could not mine the data to examine differences in practice between these specialities. This may be relevant since previous studies have reported varying frequency of engagement with bereavement practices between palliative care physicians and oncologists [13, 15]. It would also be useful to pilot the survey in order to avoid issues with misinterpretation of questions.

## Conclusions

To our knowledge, this is the first UK study to report behaviours regarding condolence letter writing amongst oncologists and palliative care physicians. Practices varied widely. Under half of participants in this study stated that they often or always sent a letter of condolence. Alternatives, such as telephone calls were not often used, and it is likely that in many instances no form of condolences were offered. Whilst some doctors feel it is better to send a standard letter to all patients, many others feel that the letter should be truly personal, or not sent at all. This is an issue that doctors feel strongly about, and it is evident that any attempt to introduce policies to standardise and reduce variance in practice would be poorly received. The study provides a platform for further research, with larger numbers of doctors across a wider range of specialities including nurses and those working in bereavement services, to provide a more generalisable picture of current practices and attitudes. Policy makers may also bear these findings in mind when considering future palliative and bereavement care policies.

## Conflicts of interest

The authors declare that they have no conflict of interest.

## Author contributions

NSV conceived the study. JH, OM, and NSV constructed the survey. Data analysis was conducted by JH and NSV. JH and NSV wrote the manuscript. All authors reviewed the final version.

## Sample of survey

### Current practices

1. **How often do you write condolence letters?**
  - Always
  - Often
  - Occasionally
2. **Why do you write condolence letters?**
  - I knew the patient or family well
  - I feel it will help the family with the grieving process/anger at losing a loved one
  - It will help me come to terms with the sense of loss
  - I feel the family would be disappointed/upset by the absence of a condolence letter
  - It is an opportunity for the family to come and talk about the case and for me to answer any questions
  - Other:
3. **Do you use a standard template for the letter?**
  - Yes (if possible, please provide examples of the wording used)
  - No
4. **Do you type or handwrite the letter?**
  - Type
  - Handwrite
5. **How do you present the letter?**
  - On headed trust paper
  - In a condolence card
  - Other, please specify:
6. **When would you typically send the letter?**
  - Within 2 weeks
  - 3–6 weeks
  - >6 weeks
  **And why?**
7. **Do you routinely use any of the following methods in conjunction with condolence letters?**
  - Telephone call
  - Visit to family home
  - Attending the funeral
  - None
  - Others:
